# On the Relative Relevance of Subject-Specific Geometries and Degeneration-Specific Mechanical Properties for the Study of Cell Death in Human Intervertebral Disk Models

**DOI:** 10.3389/fbioe.2015.00005

**Published:** 2015-02-11

**Authors:** Andrea Malandrino, José M. Pozo, Isaac Castro-Mateos, Alejandro F. Frangi, Marc M. van Rijsbergen, Keita Ito, Hans-Joachim Wilke, Tien Tuan Dao, Marie-Christine Ho Ba Tho, Jérôme Noailly

**Affiliations:** ^1^Biomechanics and Mechanobiology, Institute for Bioengineering of Catalonia, Barcelona, Spain; ^2^Center for Computational Imaging and Simulation Technologies in Biomedicine (CISTIB), Department of Mechanical Engineering, The University of Sheffield, Sheffield, UK; ^3^Orthopaedic Biomechanics, Department of Biomedical Engineering, Eindhoven University of Technology, Eindhoven, Netherlands; ^4^Center of Musculoskeletal Research Ulm, Institute of Orthopaedic Research and Biomechanics, University of Ulm, Ulm, Germany; ^5^UTC CNRS UMR 7338, Biomécanique et Biongénierie (BMBI), Université de Technologie de Compiègne, Compiègne, France

**Keywords:** poroelasticity, damage model, intervertebral disk degeneration, subject-specific modeling, disk cell nutrition, finite element modeling, Lumbar spine

## Abstract

Capturing patient- or condition-specific intervertebral disk (IVD) properties in finite element models is outmost important in order to explore how biomechanical and biophysical processes may interact in spine diseases. However, disk degenerative changes are often modeled through equations similar to those employed for healthy organs, which might not be valid. As for the simulated effects of degenerative changes, they likely depend on specific disk geometries. Accordingly, we explored the ability of continuum tissue models to simulate disk degenerative changes. We further used the results in order to assess the interplay between these simulated changes and particular IVD morphologies, in relation to disk cell nutrition, a potentially important factor in disk tissue regulation. A protocol to derive patient-specific computational models from clinical images was applied to different spine specimens. *In vitro*, IVD creep tests were used to optimize poro-hyperelastic input material parameters in these models, in function of the IVD degeneration grade. The use of condition-specific tissue model parameters in the specimen-specific geometrical models was validated against independent kinematic measurements *in vitro*. Then, models were coupled to a transport-cell viability model in order to assess the respective effects of tissue degeneration and disk geometry on cell viability. While classic disk poro-mechanical models failed in representing known degenerative changes, additional simulation of tissue damage allowed model validation and gave degeneration-dependent material properties related to osmotic pressure and water loss, and to increased fibrosis. Surprisingly, nutrition-induced cell death was independent of the grade-dependent material properties, but was favored by increased diffusion distances in large IVDs. Our results suggest that *in situ* geometrical screening of IVD morphology might help to anticipate particular mechanisms of disk degeneration.

## Introduction

Early changes in the optimal biophysics of the intervertebral disk (IVD) are thought to be one of the major causes of degeneration, in contrast to the normal aging processes (Smith et al., 2011). As such, these changes are related to the long-term pathologies of the human spine and to the derived socio-economical burden. However, the complex IVD environment is difficult to study, and the explorations of cause and effect relationships in disk degenerative disease (DDD) still needs more efforts.

For instance, the nutritional supply for disk cells has been pointed out as a possibly important factor involved in DDD (Huang et al., 2014). The IVD is the largest avascular organ in humans and the metabolism of the relatively few disk cells has to ensure the maintenance of a large amount of extracellular matrix (ECM), while it mainly relies on proper diffusion of metabolites from and to the periphery of the disk. Such a situation can have deleterious effects in the innermost regions of the disk (Huang et al., 2014), which might be worsen by the fact that disk cells seem particularly prone to catabolic responses in presence of limited nutrition (Rinkler et al., 2010; Neidlinger-Wilke et al., 2012). Yet, disk cell biology should be considered along with the mechanical competence of the disk tissues.

For example, preliminary numerical analyses (Malandrino et al., 2013) suggested that mechanical compaction of the ECM could alter the metabolic transport throughout the disk, being this alteration a possible predominant cause of cell death under mechanical overloads. Reported as “indirect mechanotransduction,” this phenomenon has been pointed out as a possible important source of disturbance for ECM maintenance (Iatridis et al., 2006). However, the interplay between cell nutrition and IVD mechanics is difficult to explore in an integrated way.

Among all research techniques, numerical modeling stands for a promising tool to better understand the complex mechanobiology of the musculoskeletal system (Halloran et al., 2012; Henak et al., 2013). Existing finite element (FE) spine models revealed a growing capacity to integrate coupled effects of mechanical and biophysical phenomena, and of specific tissue and geometrical properties (Noailly and Lacroix, 2012). While the relation between spine biomechanics and implants has been an important target, the possibility of investigating the effect of non-mechanical factors in DDD and regenerative therapies is another major advantage of numerical models (Noailly et al., 2014).

Focusing on the important question whether nutrition could be a relevant actor in IVD pathogenesis, several FE models have been created in order to explore the implication of indirect mechanotransduction under different simulated loads and tissue conditions. Among the different results achieved, it was found that mechanical deformations might favor cell nutrition as long as disk tissue properties remain healthy (Malandrino et al., 2011). In contrast, FE analyses have suggested that tissue degenerative changes might induce nutrition-related cell death under physiological load magnitudes, when the boundary supply of nutrients is reduced, e.g., through hypothetical endplate sclerosis (Zhu et al., 2012; Malandrino et al., 2014b). As for treatment therapies, coupling cell nutrition-poro-mechanical models have been used to explore possible anabolic treatments, e.g., with growth factors, for regenerative strategies (Huang et al., 2012; Travascio et al., 2014).

Though model validation is an unresolved issue for such theoretical studies, the possibility to generate new educated guesses through FE simulations is a clear asset in order to define new experiments or alternative patient explorations/clinical result analyses. However, this asset can only be duly exploited if model interpretations are built on a sufficiently broad view of the range of results that may arise from the variability of the input hypotheses. Most of the disk poro-mechanical models coupled to cell nutrition models are based on different osmo-poro-hyperelastic formulations initially developed to simulate healthy tissues. The poro-mechanical response of degenerated tissues was then typically simulated by decreasing the porosity and the capacity to develop osmotic pressure, and by increasing the stiffness constants (Malandrino et al., 2011; Zhu et al., 2012). However, these models of degenerated tissues have never been validated to our knowledge. Disk degeneration involves proteoglycan depletion, a reduced production of collagen II that competes with tissue fibrosis, and cracks occurrence (Antoniou et al., 1996, 2013). Hence, the multiphysics changes that occur in degenerating tissues are complex, and might be difficult to capture only through simple parameter value adjustments. As such, we raise the question whether a poro-mechanical constitutive equation initially validated to simulate healthy tissues can simulate the degenerative changes cited above.

As for the geometrical effects, it has been shown that the prediction of solutes diffusion within IVD models depends on the specific geometry assumed (Motaghinasab et al., 2014). Disk height reduction has often been considered in the simulation of degenerated changes (Galbusera et al., 2011a; Malandrino et al., 2011, 2014b; Zhu et al., 2012), and evaluated as beneficial to cell nutrition (Galbusera et al., 2011a; Malandrino et al., 2014b). However, most of the models represented disk heights of about 10 mm in their healthy configuration, which is a typical lumbar disk dimension for the average population (Noailly et al., 2007), but might change patient-specifically. Actually, measuring disk height accurately is not straightforward, and results largely depend on the landmarks chosen from plane MRI or radiographs (Frobin et al., 1997; Tunset et al., 2013). Moreover, whether the respective progresses of disk height reduction and degeneration are correlated to each other has been questioned (Frobin et al., 2001). All these geometrical uncertainties about the healthy and degenerated IVD suggest that different interactions might be predicted between disk cell nutrition and disk degeneration, depending on the patient considered. Hence, quantifications of patient-specific 3D disk geometries (Neubert et al., 2014) along with multiphysics disk tissue changes appear necessary, in order to capture these interactions.

Accordingly, the present study stands for a preliminary exploration of patient- and degeneration-specific geometrical and mechanical factors in order to investigate possible onsets of disk degeneration related to cell nutrition issues. First, the ability of IVD poro-mechanical modeling to simulate disk degeneration effects was assessed. We focused on the physical significance of degeneration-dependent tissue model parameters values, achieved through an inverse optimization problem, i.e., by minimizing the differences between the predictions of specimen-specific geometrical models and *in vitro* measurements. In particular, we considered the need to simulate the effect of cracks on the calculated multiphysics response of the disk tissues. The best degeneration-specific material parameters found were used for an early examination of the relative importance of the degeneration-dependent mechanical competence of the ECM, in particular disk geometries.

## Materials and Methods

### Cadaveric specimens

Four cadaveric human lumbosacral spines (L1-S1), i.e., Sp#1, Sp#2, Sp#4, and Sp#6 were provided by the Anatomy Department of the University Medical Center Utrecht, The Netherlands (Table 1). No additional information on the medical history was provided. Postmortem, specimens were stored at −25°C until the moment of use. L1-S1 specimens were harvested including all ligaments and para-spinal muscles. Anterior–posterior and lateral digital radiographs of the specimens were used to exclude severely degenerated spines. The specimens were scanned with both MRI and qCT for both grading and reconstruction protocols. CT protocol was performed at Polyclinique Saint Côme (Compiègne, France). Moreover, they were scanned in sagittal plane using the T2-weighted MRI protocol on a 1.5 T GE MRI machine. The slice thickness was 3 mm, the matrix was 512 × 512, and the field of view (FOV) was 360 mm × 360 mm. The repetition time (TR) and echo time (TE) were 3380 and 105.588 ms, respectively. The number of images was 19 and the acquisition time was about 3 min. All IVDs of all the L1-S1 specimens were graded by an experienced radiologist in terms of degeneration Pfirrmann score (Pfirrmann et al., 2001).

**Table 1:** **Specimen information**.

Sample ID	Age, Gender
Sp#1	67, ♀
Sp#2	50, ♂
Sp#4	52, ♂
Sp#6	66, ♂

### Experimental protocol

One L1-S1 specimen (Sp#4) was tested *in vitro* under flexion–extension, lateral bending, and axial rotation (see also Validation Study). Before testing, the specimens were allowed to thaw at room temperature overnight. Then, all muscles were carefully excised to prevent damaging the ligaments and cartilaginous tissues. The L1 and S1 vertebrae were embedded in Polymethyl Methacrylate. S1 was fully constrained in the testing machine (Wilke et al., 1994). Pure unconstraint moments of 5 Nm were applied on the upper plate with a constant loading rate of 1 deg/s in flexion–extension and lateral bending and 0.5 deg/s in axial rotation. These load directions were defined as principal directions, and the ranges of motion (RoM) were measured. Previous to measurements, a cycle of 5 Nm unconstrained flexion–extension was applied at 1 deg/s in order to assess the absolute neutral position. Then, two full cycles of preconditioning were applied in each principal direction, under loading conditions similar to those used for the mechanical analysis of the specimens. No follower load was applied, nor was the pressure in the IVDs measured.

**Figure 1:**
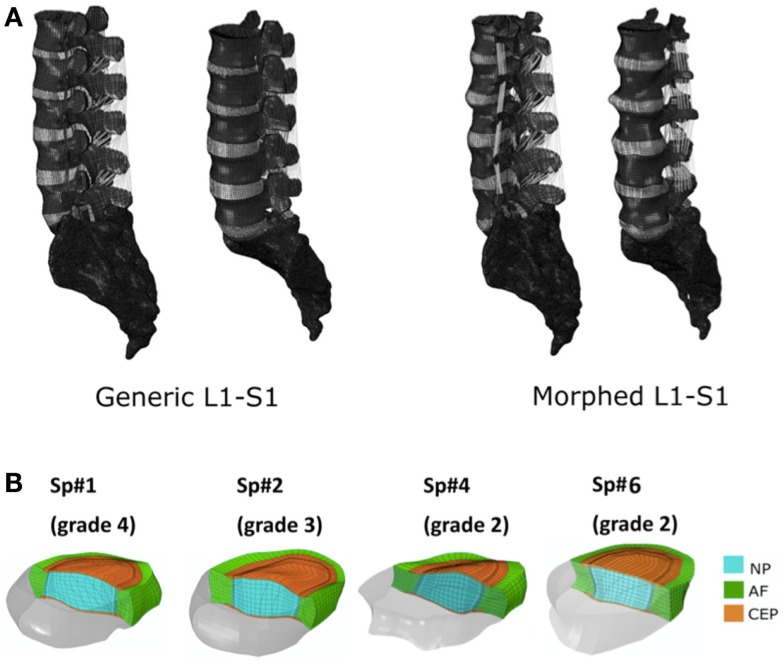
**(A)** Generic and example of a morphed FE models of the full L1-S1 osteo-ligamentous spine and **(B)** MRI-based FE reconstructions of the four L3-L4 IVDs used in the optimization study (note: the scale is not uniform from one model picture to another. Please refer to Table 5 for the dimensions).

Subsequently, creep compression experiments were performed on the L3-L4 segments of the four lumbar spines (Figure 1). The L3-L4 monosegments were excised from the L1-S1 specimens, and positioned on the testing machine in their neutral position. All degrees of freedom were fixed and locked in the gimbal system of the testing machine. Only vertical translations were allowed and measured, in response to the imposed axial compression. A compressive preload of 300 N was applied for 180 s, then released for 180 s. This cycle was repeated three times, in order to minimize the effect of IVD superhydration. Next, the load onto the spinal motion segment (SMS) was increased to 500 N in 10 s, and maintained for 3 h. The creep response was measured in terms of recorded displacements. All mechanical tests were performed in the spine-testing laboratory of the Institute of Orthopedic Research and Biomechanics (Ulm, Germany).

### FE models based on MR and qCT images

Applying the protocol developed in the framework of the EU-funded project MySpine, the cadaveric specimen geometries of lumbar vertebrae and IVDs were reconstructed from MR and QCT images, and a generic FE model geometry (Figure 1A) was morphed to the subject-specific geometries. The IVD segmentation method has been presented in Castro-Mateos et al. (2014), and the vertebra segmentation in Castro-Mateos et al. (2015). The morphed FE meshes of the IVDs were representatives of the patient-specific proportions of nucleus pulposus (NP) and annulus fibrosus (AF) as detected from the MR images (Figure 1). The specific shape of the cartilage endplate (CEP) resulted from these adaptations.

The morphing of the generic model onto the 3D geometrical reconstructions of the specimens tested followed a method, the details of which are being reported independently (Pozo et al., in preparation). Briefly, the similarity energy was composed of two symmetric terms measuring the distances between the patient-specific surface geometry and the corresponding surface on the morphed model. The internal regularization energy was based on elastic energies relative to the element edge lengths and element solid angles. The transformation was parameterized with cubic B-splines, and the optimization algorithm was gradient-descent with direct line-search. The morphing method preserved the relative sizes of the elements as well as the mesh structure at material discontinuities, as previously determined through an extensive mesh convergence analysis of the IVD generic model (Ruiz et al., 2013). Such preservation aimed to maximize the convergence and avoid as much as possible poro-mechanical instabilities under physiologic load rates. It also guarantied the non-convexity of the elements.

The model included ligaments with a non-linear elastic tensile behavior, modeled through a power law for the toe region and through a linear stress–strain relationship for the linear region of the ligaments (Noailly et al., 2012). The model has three different groups of element-type: shell elements (vertebras and sacrum), hexahedral elements (disks and facets), and truss elements (ligaments). Facet cartilages and vertebral bodies were modeled as quasi-rigid bodies (i.e., ~30 GPa Young’s modulus), and facet contact was considered frictionless and resolved through a penalty algorithm with a penalty normal stiffness of 200 N/mm (Schmidt et al., 2009).

### Constitutive modeling of the IVD

The AF and the NP were both modeled as poro-hyperelastic materials, and the CEP as a poro-elastic material. In the poro-hyperelastic materials, the total stress tensor $\underset{=}{\sigma }$ generated by external loads was the superimposition of the porous solid stress and the fluid pore pressure, *p*, which were respectively derived from a strain energy density function *W*, and from Darcy’s law:

(1) $$W=\frac{G}{2}\left(I_{1}-3\right)+\frac{K}{2}{\left(J-1\right)}^{2}+W_{\mathrm{ANI}}$$

(2) $$\underset{=}{\sigma }=\frac{1}{J}\frac{\partial W}{\partial \underset{=}{F}\;}{\underset{=}{F}\;}^{T}-p\underset{=}{I}$$

(3) $${\underline{u}}_{\text{f}}\;\phi =\underline{\underline{k}}\cdot \underline{\nabla p}$$

In the above equations, *G* and *K* are respectively the shear and the bulk modulus of the drained porous solid skeleton. *W*_ANI_ is an anisotropic strain energy density term, different from zero only for the AF (see Eq. 6). $J=\det\;\underset{=}{F}$ is the volumetric strain with $\underset{=}{F}\;$ the deformation gradient tensor of the continuum, *I*_1_ is the first strain invariant, $\underset{=}{I}\;$ is the second order unit tensor, ${\underset{-}{u}}_{f}$ is the pore fluid velocity, and ϕ and $\underset{=}{k}\;$ are respectively the porosity and the hydraulic permeability tensor of the tissue. The two phases, i.e., fluid and solid, were assumed to be nearly incompressible, and the porosity varied with the deformation of the continuum, with respect to an initial value ϕ_0_:

(4) $$\phi =1-J^{-1}\left(1-\phi_{\text{0}}\right)$$

For all tissues (AF, NP, and CEP), an isotropic hydraulic permeability was considered and depended on the initial porosity, on the volumetric strain, and on an initial permeability, *k*_0_ [5]:

(5) $$\underset{=}{k}\;=\left[k_{0}\left(\frac{\left(1-\phi_{\text{0}}\right)\phi }{\phi_{0}\left(1-\phi \right)}\right)\exp\left(\frac{M\left(J-1\right)}{2}\right)\right]\underset{=}{I}$$

being *M* an empirical coefficient.

In the AF, the anisotropic and non-linear fiber-induced strengthening was taken into account through the *W*_ANI_ strain energy density term from Eq. 1 (Gasser et al., 2006):

(6a) $$W_{\mathrm{ANI}}=\frac{K_{1}}{K_{2}}\underset{\delta =1}{\overset{2}{\Sigma }}\;\left\{\exp\left[K_{2}{\left\langle {\bar{E}}_{\delta }\right\rangle }^{2}\right]-1\right\}$$

(6b) $$\mathrm{with}\;{\bar{E}}_{\delta }=\kappa \left(I_{1}-3\right)+\left(1-3\kappa \right)\left[(a_{0{,}_{\text{δ}}}⊗\;a_{0{,}_{\text{δ}}}:\underline{\underline{C}})-1\right]$$

(6c) $$\mathrm{and}\;\left\langle {\bar{E}}_{\delta }\right\rangle =\frac{1}{2}\left(\;\left|{\bar{E}}_{\delta }\right|+{\bar{E}}_{\delta }\right)$$

This anisotropy term depended on fiber stiffness parameters, i.e., *K*_1_ and *K*_2_, and on the strain-like quantity ${\bar{E}}_{\delta }$, with *a*_0,δ_ and $\underset{=}{C}$ being the unit vector in the direction δ and the right Cauchy–Green tensor. Hence, it represented the square of the stretch, active only in two fiber directions (δ = 1,2), respectively characterized by angle values opposite in sign, according to the criss-cross distribution of the fibers in the AF. The parameter κ describes the level of dispersion in the fiber directions, varying from 0 (fully aligned fibers) to 1/3 (fully random fibers). Equation 6c imposes the condition that only fibers with positive strains contribute to the AF stiffness.

Within the NP, proteoglycan-induced NP swelling was described by considering the fluid pressure as a sum of the water chemical potential, *u*_w_, and a swelling pressure related term, ΔΠ, which was assumed constant during deformation (Wilson et al., 2005; Galbusera et al., 2011b):

(7) $$p=u_{w}+\Delta \Pi$$

### Damage modeling approach

Undamaged drained shear (*G)* and bulk (*K*) moduli were both identical for the AF and for the NP (Schroeder et al., 2010). This equality assumed that the differences in deviatoric and volumetric stiffness between the two healthy tissues arise from the differences in oriented collagen and water contents, at low strains. According to the theoretical developments proposed by Dormieux and Kondo (2007), a dimensionless damage parameter, *d*, was used to set initially the effective damaged moduli *G_d_* and *K_d_* with both AF- and NP-specific values, based on a Mori–Tanaka homogenization estimate (Mori and Tanaka, 1973) for elastic constant in a medium with open cracks:

(8) $$G_{d}=\frac{G}{1+Q_{G}d}$$

(9) $$K_{d}=\frac{K}{1+Q_{K}d}$$

where $Q_{G}=\frac{32}{45}\frac{\left(1-\nu )(5-\nu \right)}{2-\nu }$ and $Q_{K}=\frac{16}{9}\frac{1-\nu^{2}}{1-2\nu }$ were only function of the undamaged Poisson’s ratio ***ν*** of the solid matrix, assumed to be 0.2 (Ferguson et al., 2004; Johannessen and Elliott, 2005). No damage was assumed for the CEP.

Exploiting the volume-based definition of *d* as a crack density factor multiplied by the cube of a typical crack length (Budiansky and O’Connell, 1976), we further introduced a modification of this micromechanics-based approach: the density of cracks altered the initial porosity as well, assuming thus that cracks were additional cavities saturated by fluid, and connected to the rest of the pores:

(10) $$\phi_{0d}=\phi_{0}+\frac{4\mathrm{Xd}\pi }{3}$$

being ϕ_0_*_d_* the damaged initial porosity and *X* = 0.01 an aspect ratio that simulates ellipsoidal needle-shaped cracks, with a major semi axes two orders of magnitude larger that the minor semi axis.

### Optimization of the poro-hyperelastic material and damage constants

In order to obtain inversely a set of material properties as a function of the degeneration grade, the respective experimental creep responses of the three L3-L4 IVDs with degeneration grades from two to four were considered as gold standards. For this optimization study, vertebrae were not modeled, and the effect of the posterior elements on the vertical creep displacement was neglected. Thus, only the specimen-specific disk models were used, and for each IVD, an objective function was defined as the average least square difference between the experimental and the predicted creep displacements, *u*, as follows:

(11) $$S_{\mathrm{creep}}=\frac{\sum_{j=1}^{N}{\left(u_{\mathrm{EXP,}j}-u_{\mathrm{FEM,}j}\right)}^{2}}{N}$$

where *N* is the number of points considered for the optimization along the experimental creep curve. FE-predicted displacements were obtained by imposing a load of 500 N, as used experimentally, which increased linearly from 0 to 10 s (short-term compressive response) and was subsequently maintained for 3 h (long-term creep response). No preconditioning cycles were simulated: the swelling pressure value in the NP at time zero was considered as a pre-stress in the initial condition, and was kept constant for the remaining simulated time.

Before undertaking the numerical optimizations, a design of experiment (DOE) approach was used to screen out the main factors affecting the displacement response in the short-term and in the long-term. A fractional factorial design was used (Malandrino et al., 2009), and two DOE studies were performed for one specimen-specific L3-L4 model (Sp#2, grade 3): while one study allowed examining the parameters that most affected the short-term compressive, the other tackled the long-term creep response. The influence of each parameter on each response was measured with the ANOVA standardized effect (Montgomery, 2001), and the level of significance α was set to 0.01. The parameter space was defined by: the initial CEP hydraulic permeability (*k_0_*, see Eq. 5), the shear and bulk undamaged moduli (*G*, *K*, see Eqs 8 and 9), the AF and NP damage (*d*, see Eqs 8–10), the AF dispersion fiber (κ, see Eq. 6b), and the NP swelling pressure (ΔΠ, see Eq. 7). Extreme values were fixed for the exploration of the parameter space (Table 2). Values of parameters such as initial tissue porosities are already known to have an important effect on the poro-mechanical response of disk models and are unequivocally associated to disk degeneration. Hence, they were directly taken into account in the numerical optimizations without being previously explored through the DOE.

**Table 2:** **Range of values (high and low levels) for the parameters analyzed with the DOE sensitivity, and values used as components for the initial vector of parameters of the downhill simplex Python algorithm**.

Parameter	CEP *k_0_* (mm^4^/Ns)	*G* (MPa)	*K* (MPa)	AF *d*	NP *d*	κ	ΔΠ (MPa)
Range for DOE	2 × 10^−03^–2 × 10^−02^	0.5–1.0	0.5–10.0	0.0–1.0	0.0–1.0	0.0–1/3	0.0–0.2
Initial value for downhill simplex	Non-optimized	0.5	0.5	0.1	0.1	0.01	0.15
Reference	–	Fujita et al. (1997), Périé et al. (2005), Eberlein et al. (2001)	Périé et al. (2005)	Assumed	Assumed	Assumed	Johannessen and Elliott (2005), Galbusera et al. (2011b)

After having screened out the most important model parameters for both the short- and the long-term displacements, the specimen-specific optimizations were run by using the downhill simplex approach implemented in SciPy optimization toolbox (Oliphant, 2007). The initial vector to optimize was chosen based on literature data (Table 2). Since initial effective porosity ϕ_0_*_d_* was controlled through the damage parameter (Eq. 10), undamaged initial porosities ϕ_0_ in Eq. 10 were fixed. According to biochemical measurements, values were respectively set to 0.75 and 0.8 for the AF and for the NP of grade 2 IVD, and to 0.7 and 0.75 for the AF and NP of disks with higher degeneration grades (Dao et al., 2014).

Optimizations were also run with no consideration of damage in order to assess the relevance of this parameter in the constitutive behavior. In this latter case, AF and NP initial porosities were optimized as independent parameters.

### Validation study

The reliability of the optimized grade-dependent material parameters was tested through an additional validation study for the L1-S1 specimen tested *in vitro* (Sp#4). The corresponding FE morphed model was loaded under unconstrained flexion, right lateral bending, and left axial rotation with moments of 5 Nm, simulating the experimental protocol detailed above. For each IVD, material properties were introduced in the FE model as a function of the Pfirrmann grade (see Table 4 in the Section “Result”). FE calculations were compared to the experiment in terms of both global RoM (rotation of L1 with respect to S1) and segmental RoM (e.g., rotation of L1 with respect to L2, etc.).

### Mechano-transport simulations

Mechanical deformations determined changes in the amount of fluid/porosity and in the diffusion distances. A sequential mechanical-transport algorithm was used (Malandrino et al., 2011), in which the deformations from the mechanical simulation acted as template for all transport simulations. In the deformed geometry, diffusion of glucose, lactate, and oxygen were modeled through standard 3D diffusion-reaction:

(12) $$\frac{\partial }{\partial t}\left(\begin{array}{c} \;C_{{\text{O}}_{2}}\\ C_{1}\\ C_{\text{g}} \end{array}\right)-\left(\begin{array}{ccc} \;D_{{\text{O}}_{2}} & 0 & 0\\ \text{0} & \;D_{1} & 0\\ \text{0} & 0 & \;D_{\text{g}} \end{array}\right)\nabla^{2}\left(\begin{array}{c} \;C_{{\text{O}}_{2}}\\ C_{1}\\ C_{\text{g}} \end{array}\right)=\left(\begin{array}{c} \;R_{{\text{O}}_{2}}\\ R_{\text{1}}\\ R_{\text{g}} \end{array}\right)$$

where components *C_i_*, *D_i_*, and *R_i_* are respectively the concentrations, the tissue-homogenized (effective) diffusion coefficients, and the reaction terms of oxygen (*i* = O_2_), lactate (*i* = l), and glucose (*i* = g). Diffusion coefficients were strain-dependent and expressed as (Mackie and Meares, 1955; Gu et al., 2004):

(13) $$D_{i}=-\frac{\phi^{2}}{{\left(2-\phi \right)}^{2}}d_{i}$$

where *d_i_* is the diffusivity of solute *i* in water, and ϕ is the current porosity, calculated from Eq. 4.

*R_i_* terms in Eq. 12 stood for the interrelated cellular consumptions of oxygen and glucose and accumulation of lactate, proportional to the cell density ρ_cell_ at each point:

(14) $$R_{O_{2}}=-\phi \frac{7.28\rho_{\mathrm{cell}}}{S_{O_{2}}}\left[\frac{C_{O_{2}}\left(\mathrm{pH}-4\mathrm{.95}\right)}{1.46+C_{O_{2}}+4.03\left(\mathrm{pH}-4\mathrm{.95}\right)}\right]$$

(15) $$R_{1}={\text{ρ}}_{\text{cell}}e^{\left[-2.47+0.93\text{pH}+0.16C_{{\text{O}}_{2}}-0.0058C_{{\text{O}}_{2}}^{2}\right]}$$

(16) $$R_{g}=-\frac{1}{2}R_{l}$$

The *pH* was a continuous model variable, linearly related with the lactate concentration (Bibby et al., 2005; Soukane et al., 2007). Equations 14–16 were derived from *in vitro* tests on IVD cell cultures (Bibby et al., 2005). The three coupled diffusion-reaction models, defined through Eqs 12–16, were solved by the FE method through the implicit solver ABAQUS 6.12 (Simulia, Providence, RI, USA). Interrelations among Eqs 14–16 were taken into account through a finite time step approach (Malandrino et al., 2011, 2014b; Malandrino, 2012).

Exponential decays over time – starting from an initial cell density ρ_cell,0_ characteristic of each tissue – simulated cell death, depending on the pH and on the glucose concentration within the tissue:

(17) $$\rho_{\mathrm{cell}}=\rho_{\mathrm{cell,0}}e^{-\left(\alpha_{g}+\alpha_{\mathrm{pH}}\right)t}$$

where α_pH_ was −3.43 × 10^−6^ s^−1^ if the pH was lower than 6.8 and 0 otherwise. Glucose-dependent cell death was implemented according to the cell death model proposed by Zhu et al. (2012):

(18) $$\alpha_{g}=K\left(\frac{C_{g}-0.5}{C_{g}+k_{1}}-\frac{\left|C_{g}-0.5\right|}{C_{g}+k_{2}}\right)$$

This formulation ensured that cell death always occurred below the critical concentration of glucose of 0.5 mM (Horner and Urban, 2001). Parameters *K*, *k*_1_, and *k*_2_ in Eq. 18 were taken directly from Zhu et al. (2012). The cell death model that further accounted for the contribution of α_pH_ (Eq. 17) was verified against its ability to reproduce temporal and spatial viability curves obtained *in vitro*, as a function of the cell densities characteristic of the NP and AF tissues, i.e., from 4 to 9 million cells/mL (Maroudas et al., 1975), and for the time periods used in the present study (0–3 days): a 26 mm width diffusion chamber – filled with cells embedded in 1% agarose gel – was simulated according to experimental tests on bovine nucleus cell viability (Horner and Urban, 2001). Oxygen, lactate, and glucose diffusivities accounted for gel porosity. Only half of a thin slice was reproduced due to chamber geometry. Initial conditions were applied throughout the chamber for the three metabolites to reproduce the experiment: an initial pH of 7.4 (i.e., an initial nil lactate concentration), an initial oxygen pressure of 21 kPa, and an initial glucose concentration of 5 mM. During simulations, these values were maintained only at the boundary since nutrient concentrations were maintained in the medium along the experiment (Horner and Urban, 2001).

For the mechano-transport simulations in the IVD models, a simplified daily load sequence was simulated, consisting in 8 h of 150 N compression (resting phase in a supine position) and 16 h of 750 N compression (physical activity phase). These levels of loads were chosen based on averages of activity and resting intradiscal pressures measured *in vivo* (Wilke et al., 1999). In order to avoid biases due to the transient initialization of the mechano-transport model, a preconditioning period of 3 days was simulated, both in terms of mechanical response and solute transport. Three additional days were subsequently simulated for cell viability calculations and for the analysis of the results. Solute boundary conditions were applied at the external CEP and AF edges with averaged values related to normal concentrations of metabolites in blood (Maroudas et al., 1975; Mokhbi Soukane et al., 2009).

The relevance of the particular IVD geometry combined to tissue mechanical competence was assessed by comparing the solute transport and cell viability results, respectively obtained with a grade 2 (Sp#4) and with a grade 4 (Sp#1) specimen-specific L3-L4 disk model. Also, a L5-S1 disk graded 4 from Sp#6 was analyzed. In all cases, grade-matching mechanical properties were used for both the AF and the NP, based on the constitutive parameter values extracted from the optimization previously described (see Results in Table 4). In order to assess further, the sole effect of mechanical competence, the grade 4 L3-L4 disk of Sp#1 was artificially modified into a “healthy” grade 2 disk in terms of material properties, and results were compared to those obtained with the grade-matching mechanical properties.

## Results

### Creep experiments L3-L4

**Figure 2:**
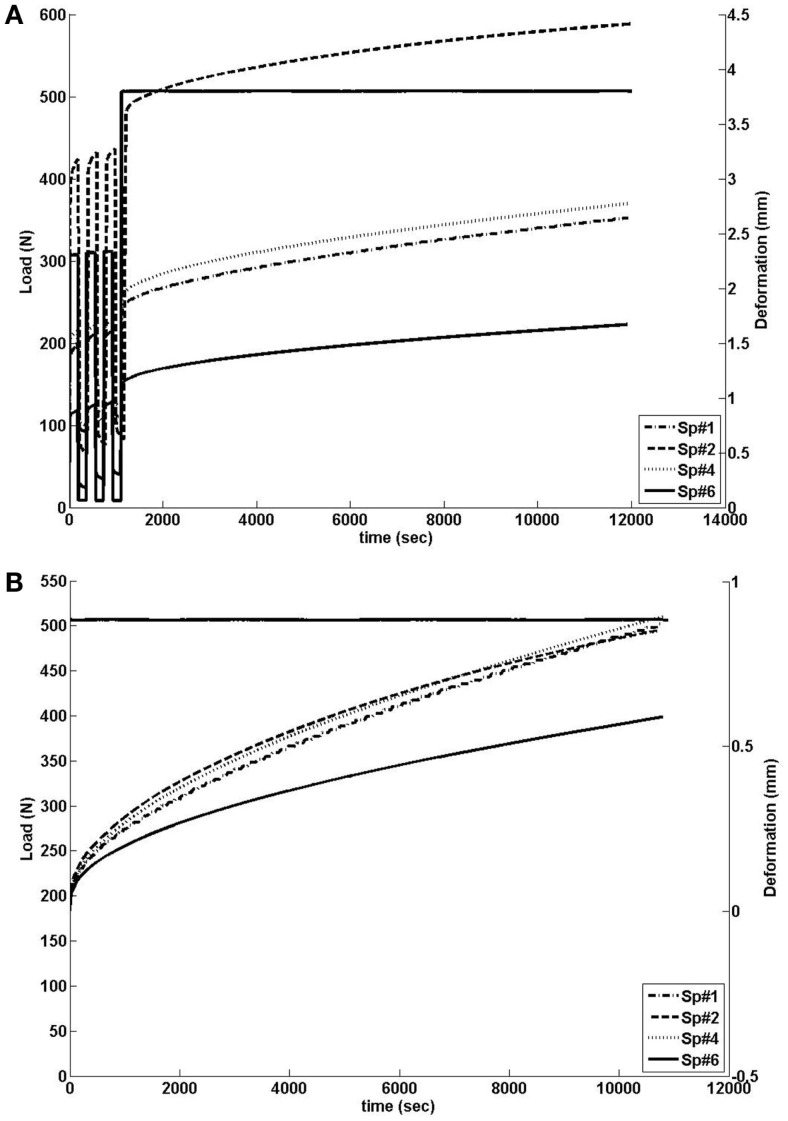
**(A)** Load history (left scale) and outcome of creep tests on the L3-L4 monosegments as measured in terms of axial displacements of the L3 vertebra (right scale). **(B)** Load history (left scale) and height loss when the effect of preconditioning was excluded (right scale).

The behavior of the four specimens was different in absolute displacement, predominantly caused by the three preconditioning cycles (see Figure 2A). However, when looking at the height loss (0.87 mm) during the actual creep test, the outcome in displacement and time-dependent behavior was similar, except for Sp#6 (see Figure 2), which had a reduced initial height compared to the other disks (See dimensions reported in Table 5). All in all, the disk specimens reached about 20% of axial deformation with respect to their central height, except the large Sp#1 disk, the central part of which deformed by about 30%.

### Optimization of the poro-hyperelastic material properties with no damage

Due its reduced height, Sp#6 disk model had extremely large element distortions and convergence issue impeded to achieve a successful optimization, independently of the constitutive material formulation. For the rest of disk specimen models, when damage was not considered (i.e., *d* = 0 in Eqs 8–10), the shear modulus increased with the degeneration grade. There were no other tendencies in terms of optimized parameters as a function of degeneration (Table 3).

**Table 3:** **No damage: optimized values as a function of the degeneration grades of the L3-L4 disks (see Table 1 and Figure 1 for specimen details) obtained through the minimization of the objective function (Eq. 11)**.

	CEP *k_0_* (mm^4^/Ns)	*G* (MPa)	*K* (MPa)	AF ϕ	NP ϕ	κ	ΔΠ (MPa)
Grade 2	2.8 × 10^−03^	0.037	1.4	0.67	0.93	0.16	0.15
Grade 3	1.0 × 10^−03^	0.32	0.52	0.98	0.87	0.13	0.001
Grade 4	3.1 × 10^−03^	0.3	0.93	0.82	0.98	0.15	0.14

### DOE sensitivity results

**Figure 3:**
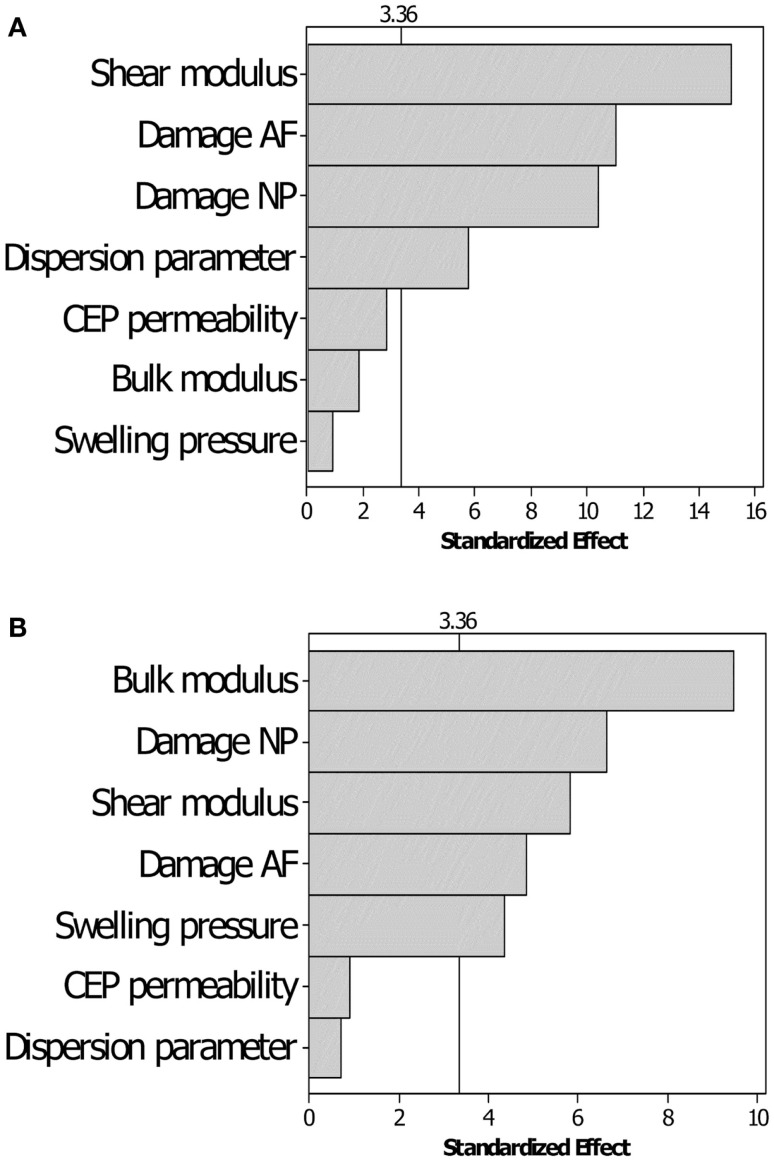
**Pareto chart for the sensitivity of the displacement responses to the variation of the parameters chosen**. **(A)** Short-term displacement and **(B)** long-term displacement responses. The threshold of significance (vertical line) for the ANOVA analyses is set to α = 0.01.

The DOE results indicated that among the seven parameters chosen, only CEP initial permeability could be excluded for further optimization, because its low significance on both the short- and the long-term displacement response (Figure 3). For the short-term simulations, the shear modulus, together with the NP and AF damage, and the dispersion parameter for AF fibers were the most important factors. For the long-term simulation, the AF dispersion parameter lost its significance, whereas the NP swelling pressure and the matrix undamaged bulk modulus had a substantially increased significance, though they were poorly significant in the short-term.

### Optimization of the poro-hyperelastic material properties with damage

**Figure 4:**
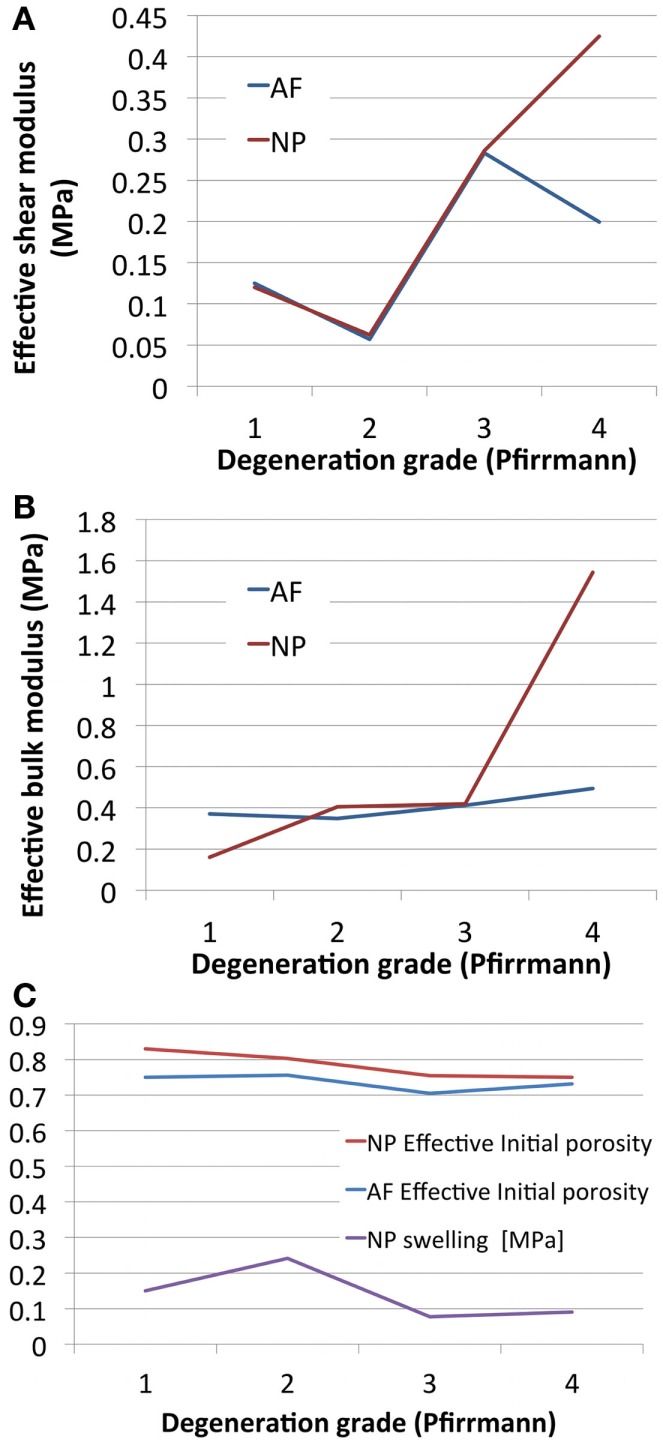
**Degeneration-dependent values of (A) the effective shear moduli, (B) the effective bulk moduli, and (C) the initial porosities and NP swelling pressures obtained after optimization**.

Optimized undamaged shear and bulk moduli both increased with the degree of degeneration. Damage *d* increased with degeneration in the AF, and decreased in the NP. NP swelling decreased with degeneration (Table 4). NP damaged (or effective) shear modulus, calculated from Eq. 8 increased with degeneration while AF damaged shear modulus showed a decrease from grade 3 to grade 4 (Figure 4A), in agreement with the increase of AF damage. Both AF and NP, calculated with Eq. 9, increased with degeneration, although to a higher extent for the NP (Figure 4B). Initial damaged AF porosities slightly decreased with degeneration (Figure 4C) and NP swelling pressure switched from approximately 0.15 MPa to values reduced by about 40–50% (Table 4; Figure 4C).

**Table 4:** **Damage: optimized values as a function of the degeneration grades of the L3-L4 disks (see Table 1 and Figure 1 for specimen details) obtained through the minimization of the objective function (Eq. 11)**.

	*G* (MPa)	*K* (MPa)	AF *d*	NP *d*	κ	ΔΠ (MPa)
Grade 2	0.069	0.487	0.139	0.071	0.006	0.241
Grade 3	0.333	0.548	0.116	0.108	0.011	0.077
Grade 4	0.425	1.543	0.746	0.000	0.000	0.091

*CEP permeability was not optimized due to the DOE results*.

**Figure 5:**
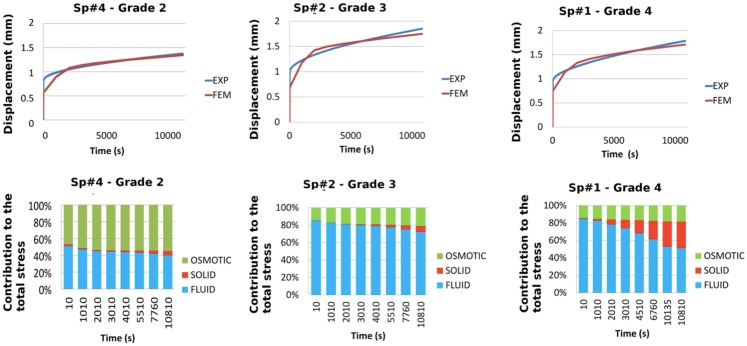
**Comparisons between experiments and FE predictions in term of total vertical creep displacement for each of the optimized L3-L4 disks (top)**. Respective contributions of the osmotic, solid skeleton, and fluid stresses to the total stress in the central NP, predicted over the duration of the creep experiment for each of the optimized disks (down).

In Figure 5, the global performance of the set of material properties reported in Table 4 for the three disks optimized is shown. When compared to the experimental outcomes, the error was lower at the end of the long-term creep and higher at the end of the short-term creep (Figure 5). Figure 5 also shows that the importance of the osmotic pressure decreased from grade 2 to grade 4 (as also derivable from the optimized values of swelling pressure in Table 4), while the importance of the solid stress with respect to the fluid stress increased with the degeneration grade.

### Validation study

**Figure 6:**
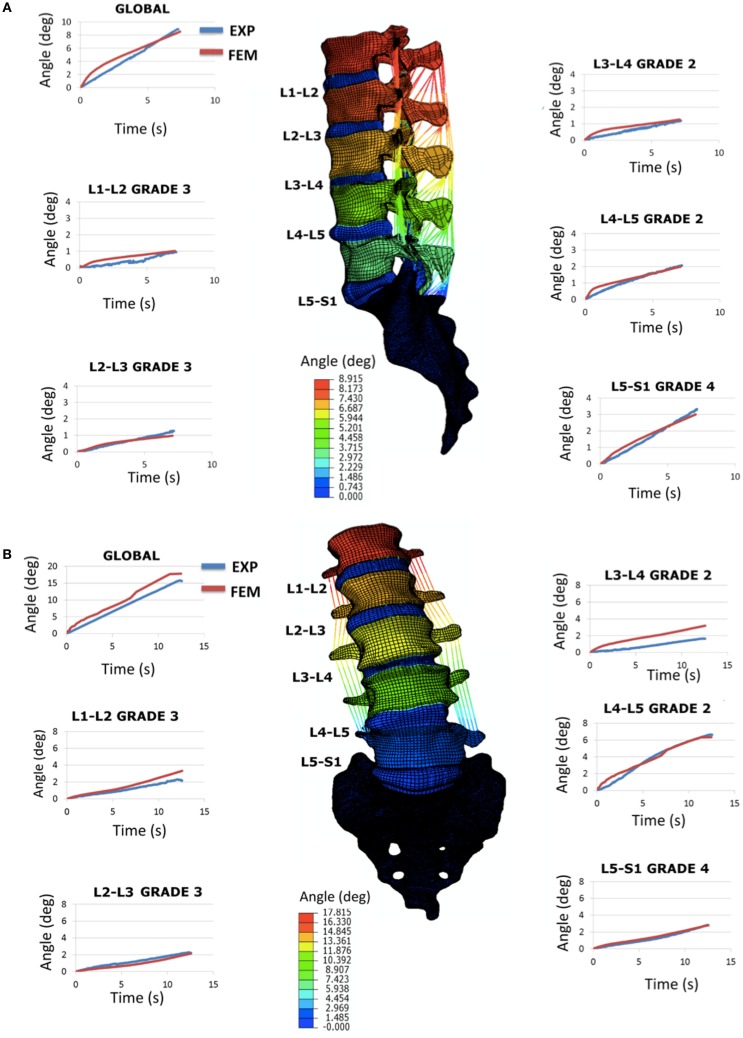
**Validation study by using the full L1-S1 FE model: (A) flexion results in terms of RoM (sagittal plane rotations in degrees) and comparison with the experimental response and (B) right lateral bending results in terms of RoM (frontal plane rotations in degrees) and comparison with the experimental response**. All the curves shown are over time (transient response) and represent both the global (L1 vs. S1) and the segmental (e.g., L1 vs. L2, etc.) rotations.

When incorporating specimen-specific distributions of the grade-dependent disk properties, the L1-S1 FE model morphed to the Sp#4 specimen geometry reproduced acceptably most of the *in vitro* flexion and right lateral bending RoM of the specimen, both globally and per segment (Figure 6). Under lateral bending, through the global RoM as well as the local RoM of three segments over five could be reproduced, the maximum rotations predicted for the L1-L2 grade 3 and for the L3-L4 grade 2 IVDs were about 40% larger than the experimental measures. As for the axial rotation load scenario, experimental results could not be reproduced by the FE predictions (data not shown, see Discussion).

### Mechano-transport simulations

**Figure 7:**
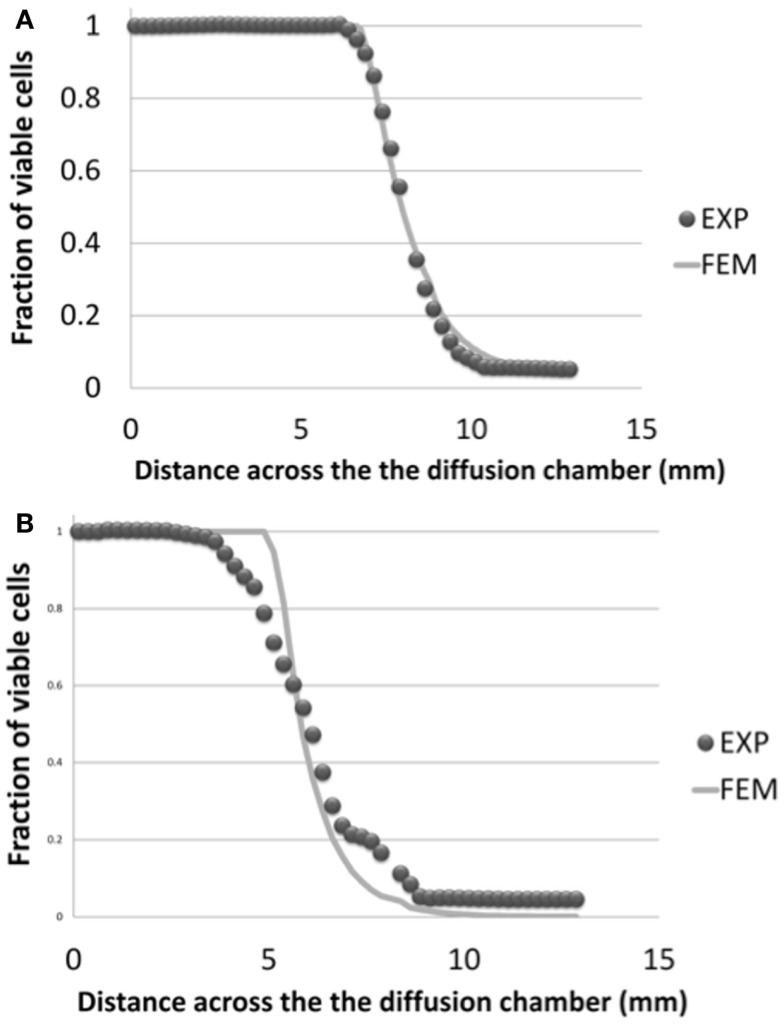
**Cell viability profiles calculated in the simulated half-slice of the diffusion chamber and comparison with experimental results (Horner and Urban, 2001), for the two cell density levels relevant to the AF and NP tissues, i.e. (A) 4 million cells/mL and (B) 8 million cells/mL**. Both numerical results and experimental data were considered at day 3 of culture.

Figure 7 shows the viability profiles predicted by the diffusion chamber model in comparison to the related experimental data (Horner and Urban, 2001), for the two cell density levels relevant to the IVD, and at the specific time relevant to the present study (3 days of culture). In terms of distance to the solute source at which cells started to die, the computed cell viability profiles were in good agreement with the experimental data.

**Figure 8:**
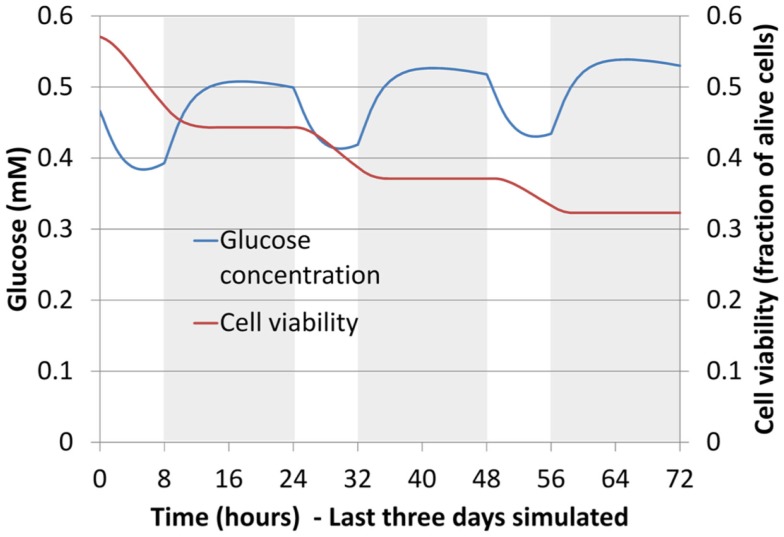
**Glucose concentration and cell viability in the AF (boundary between the inner AF and the outer AF) for largest specimen studied, in terms of height (Table 5) (Sp#1, grade 4)**. Curves are shown over time for the last 3 days of mechano-transport simulation. Shaded areas correspond to the daily activity (16 h under 750 N compression) and non-shaded ones to the rest (8 h under 150 N compression).

When 6 days of mechano-transport (including the 3 days of preconditioning) were simulated, results in terms of glucose, lactate, oxygen, and cell viability depended strongly on both the patient-specific IVD geometry, and the degeneration-specific material properties. Only cell viability results are shown (Table 5). In the Sp#1 disk model, glucose levels dropped periodically below the critical concentration of 0.5 mM, and cells kept on dying over the 3 days of cell viability simulation, though progressively slower and slower along time (Figure 8). Surprisingly, when changing the material properties of Sp#1 disk model from grade 4 (original grade) to grade 2 (altered grade), the cell death area increased (Table 5).

**Table 5:** **Results of the mechano-transport simulations for different combinations of specimen-specific geometries and degeneration-specific tissue properties, and dimensions of the different disk geometries**.

Cell viability			
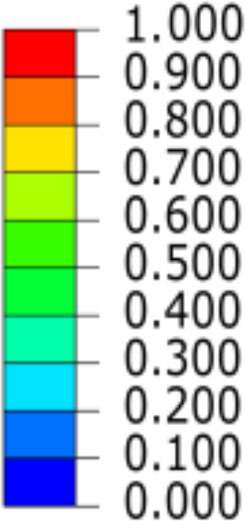	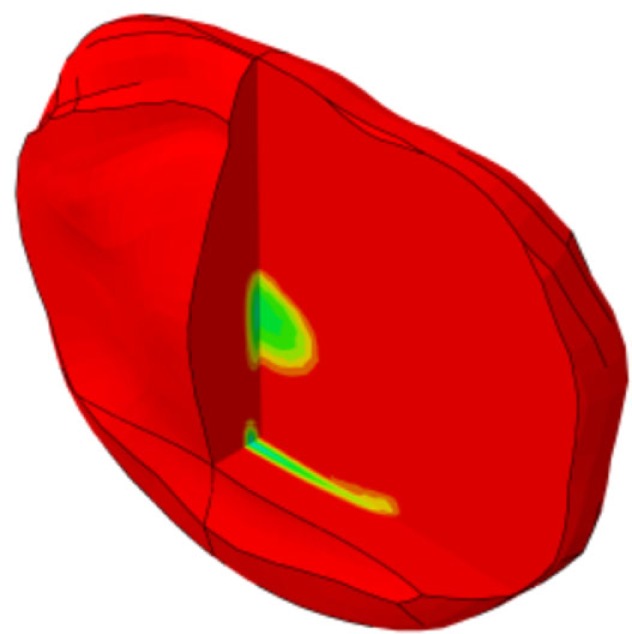	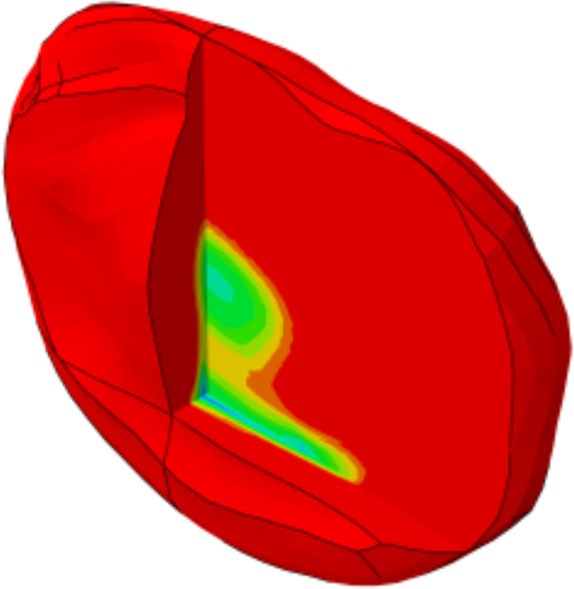	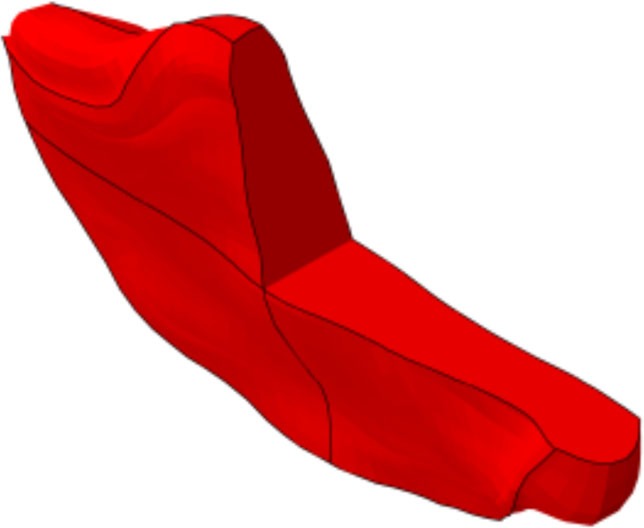	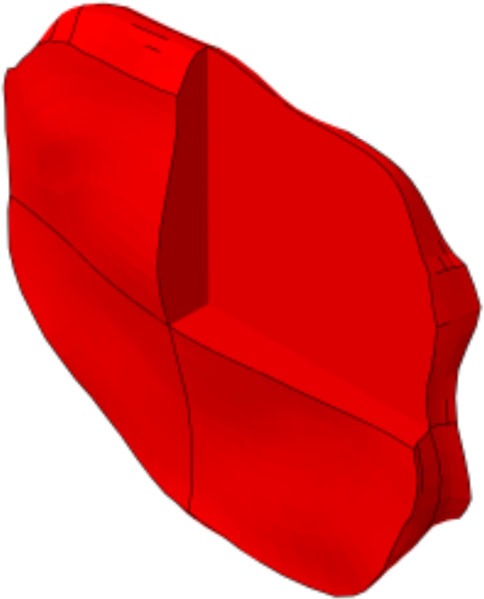

			Only 2 cutting planes are shown due to irregularity of the model

Specimen – disk level	Sp#1 – L3-L4		Sp#6 – L5-S1	Sp#4 – L3-L4

Geometry:	Grade 4	Grade 4	Grade 4	Grade 2

Material properties (Table 4; Figure 4)	Grade 4	Grade 2	Grade 4	Grade2

Distances (mm):	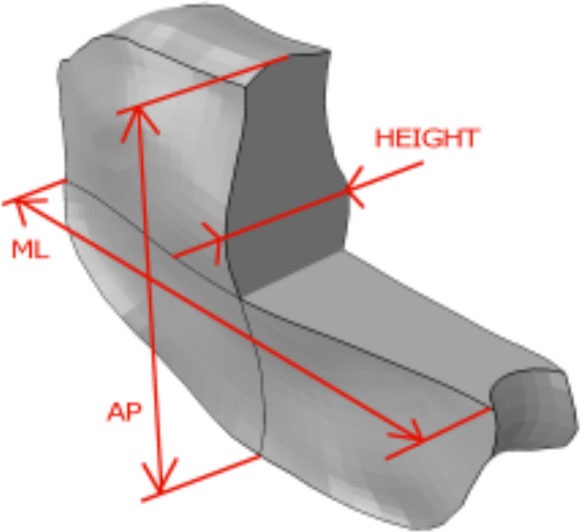			

Height	15.2	15.2	8.8	12.3
AP	38.4	38.4	38.3	39.5
ML	48.3	48.3	55.5	47.4

*Geometries are classified in function of the degeneration grade of the modeled disk specimen, and model dimensions are given. Cell viability plots correspond to the predictions achieved at the end of the simulated period (3 days of preconditioning plus 3 days of mechano-transport)*.

## Discussion

### Validity of the FEM subject-specific geometry

The correctness and accuracy of the complete subject-specific FEM models obtained after the morphing of the general model into the segmented geometries have been assessed by visual comparison and overlapping with the corresponding MRI images. In addition, the accuracy of the IVDs and the vertebrae segmentation was validated in Castro-Mateos et al. (2015), resulting in very good sub-voxel accuracies. Nevertheless, the craniocaudal concave shape of the anterior external AF of the Sp#6 disk model (Figure 1B) added to excessive element distortion affected the convergence and the capacity of the FE model to be successfully optimized. Such outcome might have arisen from a case-specific difficulty to define properly the AF external surface during the segmentation process.

### Optimization of the poro-hyperelastic material properties with no damage

Although the objective function was minimized and the inverse calculation successfully retrieved a set of parameters (Table 3), the behavior of these optimized parameters was not physically satisfactory. In particular, it has repeatedly been reported that porosity (tissue water content) of both AF and NP decreases with degeneration (Iatridis et al., 1997, 1998; Antoniou et al., 2013). This behavior was not observed for optimized parameters. Instead, very high and unrealistic porosities values were obtained for grade 3 and grade 4, probably indicative of numerical artifacts. Also, swelling pressure was at normal levels in the grade 4 IVD, in disagreement with the well known loss of proteoglycans with degeneration (Urban and Maroudas, 1981; Bibby et al., 2001). Other parameters, such as the CEP initial permeability and the AF fiber dispersion seemed to have a random effect on the optimization outcomes. These outcomes raised questions about the net effect and the physical significance of each constitutive parameter, and justified a DOE sensitivity analysis further performed with the additional inclusion of tissue damage.

### DOE sensitivity

Design of experiment sensitivity results pointed on the relative importance of the shear and bulk moduli in short- and long-term responses, respectively. Also, the significance of the damage parameter proved the phenomenological importance of this parameter, because of a direct relation to the effective elastic response of the tissue (see Eqs 8 and 9). Moreover, through the introduction of the possible presence of cracks, this approach paves the way for future studies targeting mechanistic-based description of degenerated and damaged disks. The DOE analyses also pointed out the dispersion parameter as an important factor in the anisotropic hyperelastic continuum model used (Gasser et al., 2006), under rapid loading. Though these sensitivity results correspond to a specific IVD, they were backed up by DOE analyses performed for other IVD specimen models and that included additional parameters, such as the AF permeability (data not shown).

Hydraulic permeability of the IVD tissues have been indicated as relevant parameters in other theoretical studies (Chagnon et al., 2010; Nikkhoo et al., 2013), while it has been shown that experimental AF permeability are not significantly affected by degeneration (Iatridis et al., 1998). Our sensitivity study prior to optimization confirmed these experimental outcomes and led us to exclude the CEP (and AF) permeability from the optimization studies. This choice was a compromise between the minimization of the parameter space and the accuracy of the results. However, not optimizing hydraulic permeability could be the cause of the lower optimization performance in short-term creep (Figure 5), and future optimization studies with an increased number of experimental constraints should overcome this limitation. Yet, having introduced a damage that relates with the porosity of a representative volume element (Eq. 10), also accounts indirectly for variations of the (effective) porosity-dependent hydraulic permeability (Eq. 5).

### Optimization of the poro-hyperelastic material properties with damage

Optimized poro-hyperelastic parameters combined with the concept of cracked continuum (damage) showed much more pronounced and meaningful trends than when damage was not considered (Table 3 vs. Table 4). Indeed, the increase of both the undamaged shear and bulk moduli correlates with reported fibrosis (Coventry et al., 1945; Iatridis et al., 1998; Pokharna and Phillips, 1998). Decrease of overall disk porosity and swelling pressure in the NP correlated with reported loss of fluid and drops in proteoglycans content. It could be argued that the number of specimens was limited and no statistical meaning can be attributed to the results. To this respect, further experimental–numerical explorations are planned for a next study. In the meantime, the high value of damage in the AF for severely degenerated disks (grade 4), shown in the present report, correlated very well with the presence of cracks often reported in MRI-based studies (Hilton et al., 1980; Munter et al., 2002; Sharma et al., 2009; Antoniou et al., 2013). These cracks in the AF would result in a net reduction of the stiffness of the disk. This reduction was captured by our description (Figure 4A) and has been reported *in vitro* comparing moderately (grade 3) and severely (grade 4) degenerated IVDs compressive responses (Panjabi et al., 1984; Gardner-Morse, 2004). In the NP, the nil damage prediction for grade 4 disks is obviously difficult to defend: it could indicate a numerical artifact related with the minor importance of the effective stiffness of the NP (compared to that of the AF) in controlling the global IVD vertical displacement (Malandrino et al., 2009) in severely degenerated IVDs. Also, fissures in the NP are less meaningful than those in the AF due to the relatively minor role of the NP shear resistance. As for the components that affect the effective volumetric stiffness of the NP, the model acceptably captured the reduced swelling pressure and hydration in the optimized grade 4 disks. Note that variations between grade 1 and grade 2 need to be interpreted carefully, since grade 1 properties were subjectively chosen among the data available from the literature.

All in all, optimized models reproduced nicely the creep behavior of the mono-segment specimen, in a general way. Analyzing the detail of the results, while models reproduced adequately the experimental axial displacements in the long-term, the constitutive description did not allow reproducing accurately the short-term creep experimental behavior. This could be ascribed to the absence of any explicit viscoelastic description for the collagen fibers. Nevertheless, this poro-hyper-viscoelastic description (Wilson et al., 2006) would have increased the total number of parameters, and it will be the subject of further evaluations in future studies. The solid phase contribution to total stress was shown to increase considerably when degeneration passed from grade 3 to grade 4. According to the known degeneration changes, our result illustrate increased fibrosis in the tissue, and importantly, a decreased capability of the fluid phase in the NP in bearing the load in grade 4 disks, as highlighted by other poro-elastic parametric studies (Chagnon et al., 2010).

When introduced in a whole L1-S1 FE model, the optimized degeneration-specific parameters of the disks, were able to reproduce most of the experimental responses of the specimen in terms of frontal and sagittal RoM. Notably, predictions were very accurate over time, at the global level (Figure 6). At the local level, two disk models resulted over-compliant compared to the measurements in lateral bending. Also, the FE predictions in terms of RoMs under axial rotation poorly compared with the experimental ones (results not shown). In the specific case of axial rotation, the following issues could have affected the outcomes of the comparisons: (i) the measured segmental ranges of motion were very low, hence indicating large probabilities of measurement errors; (ii) accordingly, the sum of the different intersegmental axial rotations did not correspond to the global amount of axial rotation (while it did for flexion and lateral bending loading scenarios). Thus, additional axial rotation experiments would be necessary to clarify this. But more generally, both axial rotation and lateral bending involve important coupled rotations that largely depend on the regional properties of the IVD, especially of the AF (Noailly, 2009; Noailly et al., 2011). Hence, local refinements of the disk properties might be planned based on a second step of optimizations that would use the rotation experimental data. According to the results of the present study, and to previous findings in spine model calibration (Schmidt et al., 2006), the optimized parameters should ensure a suitable model preconditioning for such a second optimization.

### Mechano-transport simulations

Mechano-transport simulations showed that disk morphology was related to the onset of cell viability. In particular, disk height controlled the diffusion distances from the source of nutrients at the endplates. This distance-related cell death appeared as the most important factor in the present study, since it could explain by itself cell death in the IVD reconstructions tested with patient-specific geometry and different sets of material properties. Nevertheless, material properties influenced the mechano-transport results as well. For example, for the specific geometry of the Sp#1 model, initially grade 4, the introduction of grade 2 properties led to an increased consolidation, i.e., increased water loss and decreased solute diffusion (Eq. 13), which exacerbated cell death occurrence. Truly, associating grade 2 properties to a grade 4 disk that might already have reduced disk height could be expected to bias the interpretation of the calculations. Hence, we compared the consolidation of disks with large (Sp#1) and small (Sp#6, Sp#4) heights, for a similar set of material parameters and similar boundary loads. We found that the larger disk led to only slightly increased tissue consolidation compare to a small disk (small in terms of disk height), i.e., 2–3% of loss of volumetric fluid fraction in the critical zone of cell death during a daily cycle. According to Eq. 13, this loss of porosity would only slightly affect the diffusivity. Thus, the consolidation-dependent diffusion appeared as a minor effect compared to the large diffusion distance effect.

In previous mechano-transport models of the IVD, mechanical loads representative of daily activity induced fluctuations of glucose concentration and cell viability but did not cause cell death *per se* in disk models with a height of about 10 mm (Zhu et al., 2012; Malandrino et al., 2014b). These studies concluded on the paramount importance of endplate-related issues, such as calcifications or decrease in vascular according to other reported simulation outcomes (Galbusera et al., 2011a; Zhu et al., 2012; Malandrino et al., 2014a). Only in such cases, increased tissue dehydration/consolidation was reported to be an additional risk factor for cell death, related to tissue degeneration (Zhu et al., 2012; Malandrino et al., 2014b). In the present study, disk size-related diffusion distance emerges as an additional relevant factor to nutrition-induced cell viability, independently on tissue degeneration. This outcome is nicely supported by experimental measurements that negatively correlated NP cell density with disk height (Rodriguez et al., 2011), and naturally follows the conclusions of recent numerical explorations of the effect of disk size on the transport of externally administered drugs (Motaghinasab et al., 2014). These concerns can now be targeted patient-specifically from diagnostic images, and could highly impact the prognosis of DDD, including explanations of gender-related risk factors (males may have larger disks, in general).

## Significance

On one hand, this study assessed the capacity of commonly used poro-mechanical disk tissue models to simulate both healthy and degenerated tissues. We found that the basic (osmo)poro-hyperelastic constitutive formulation was not able to capture simultaneously the respective effects of tissue dehydration, fibrosis, and crack occurrence. However, the introduction of a damage criterion that affected in a coordinated way the shear and the bulk stiffness, and the porosity, based on micromechanics rationales, allowed both successful simulation of known degenerated effects, and validation of the disk organ models. Coupling this new mechanical formulation to different specimen-specific IVD models confirmed the previously reported idea that AF and NP tissue degeneration alone might not cause nutrition-induced cell death, especially if cracks occur and can be filled with water. However, simulations suggested for the first time that large IVDs with healthy tissues might be particularly prone to degeneration because of cell nutrition issues mostly related to increased diffusion distances. This outcome strongly suggests that early degeneration might be related to disk morphology, i.e., a genetic characteristic unavoidably coupled to the spine mechanics.

## Conflict of Interest Statement

The Associate Editor Fabio Galbusera declares that despite being affiliated with the same institution as the author, Hans-Joachim Wilke, the review process was handled objectively and no conflict of interest exists. The authors declare that the research was conducted in the absence of any commercial or financial relationships that could be construed as a potential conflict of interest.
